# Supplementary material for: Efficacy of a Combination Therapy with Laronidase and Genistein in Treating Mucopolysaccharidosis Type I in a Mouse Model
Source: Int J Mol Sci. 2024 Feb 17;25(4):2371. doi: 10.3390/ijms25042371 (PMC10889377; doi:10.3390/ijms25042371)
Supplement: Supplementary file 1 [file ijms-25-02371-s001.zip › ijms-2821404-supplementary.pdf]

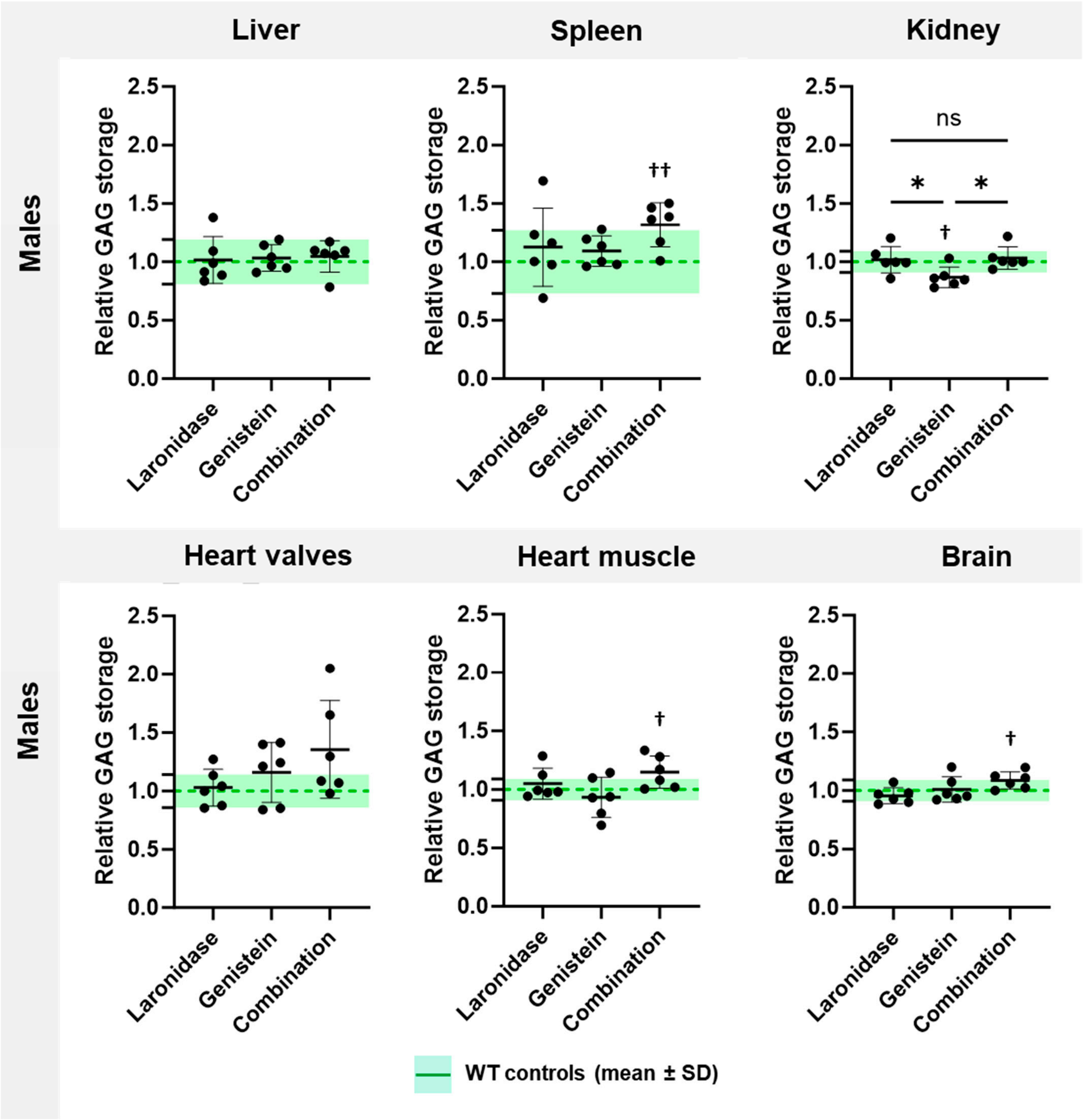

Figure S1. GAG storage in organs of 20-week-old male wild-type (WT) mice receiving laronidase, genistein, and combination therapy. GAG levels were determined in the liver, spleen, kidney, heart valves, heart muscle, and brain of WT mice after 12 weeks of treatment with 100 U/kg/week laronidase, 160 mg/kg/day genistein, or a combination of both. Results are presented as values normalized to those of untreated WT control mice (green horizontal line with standard deviation shown as shaded field). Graphs represent the results obtained for individual animals and the mean ± standard deviation. $N = 6$ animals per group. Significance: * $p < 0.05$, between treatments (Tukey's post hoc test after ANOVA); † $p < 0.05$, or †† $p < 0.01$ between treated and untreated WT animals (Student's t-test); ns—not significant.

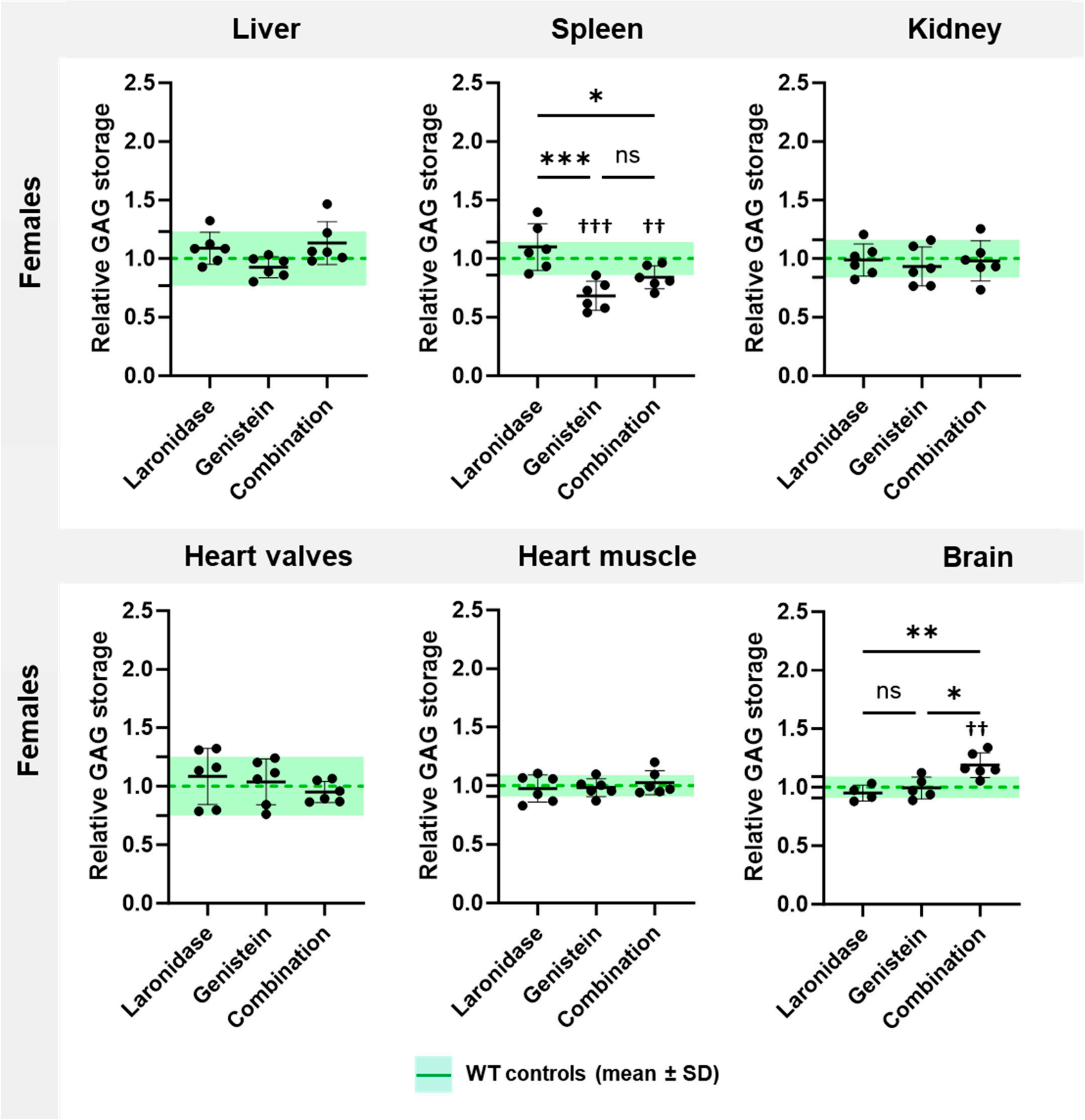

Figure S2. GAG storage in organs of 20-week-old female wild-type (WT) mice receiving laronidase, genistein, and combination therapy. GAG levels were determined in the liver, spleen, kidney, heart valves, heart muscle, and brain of WT mice after 12 weeks of treatment with 100 U/kg/week laronidase, 160 mg/kg/day genistein, or a combination of both. Results are presented as values normalized to those of untreated WT control mice (green horizontal line with standard deviation shown as shaded field). Graphs represent the results obtained for individual animals and the mean ± standard deviation. *N* = 4-6 animals per group. Significance: * $p < 0.05$, ** $p < 0.01$, or *** $p < 0.001$ between treatments (Tukey's post-hoc test after ANOVA); †† $p < 0.01$ or †† $p < 0.001$ between treated and untreated WT animals (Student's *t*-test); ns—not significant.

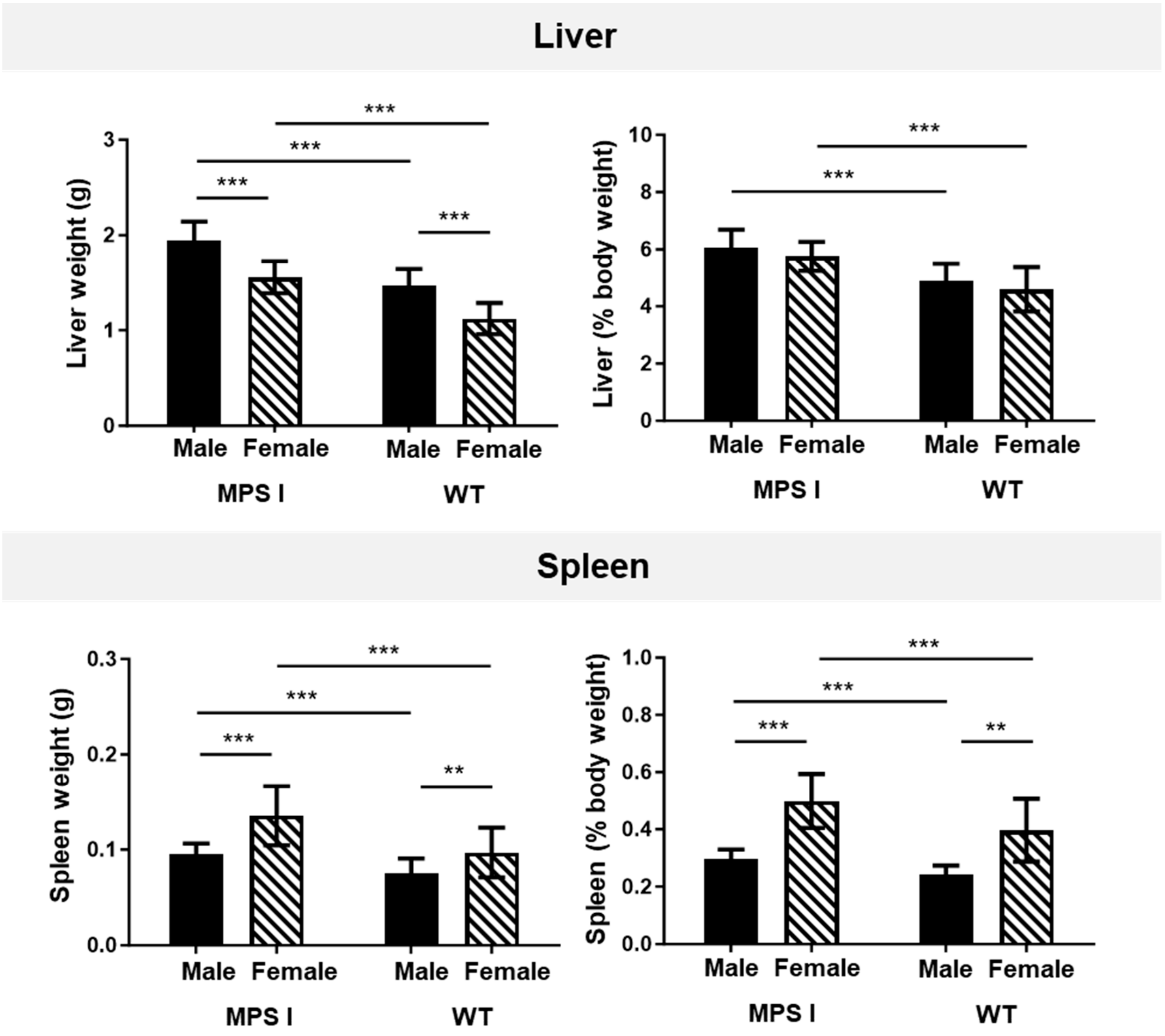

Figure S3. Liver and spleen weights of MPS I and wild-type (WT) mice. Weights were measured in 20-week-old mice of both sexes and presented as absolute organ weight (g) and organ weight relative to body weight (% body weight). Data are presented as the mean ± standard deviation. $N = 18$ animals per group. Significance: ** $p < 0.01$ or *** $p < 0.001$ between groups (Student's *t*-test).

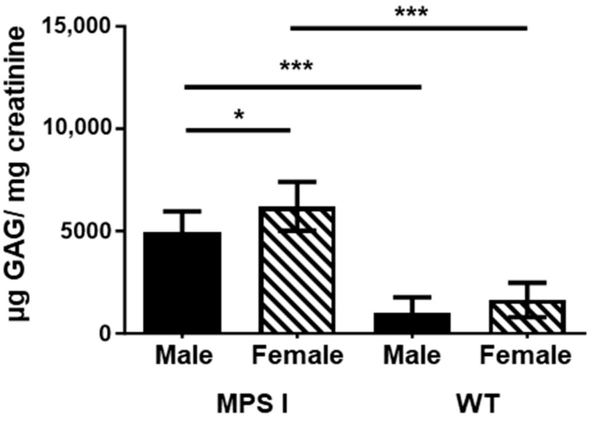

Figure S4. Total urinary GAG excretion in 20-week-old MPS I and wild-type (WT) mice. The GAG level was normalized to the urinary creatinine level. The results are presented as the mean ± standard deviation. $N = 18$ animals per group. Significance: * $p < 0.05$ or *** $p < 0.001$ between groups (Tukey's post-hoc test after ANOVA).

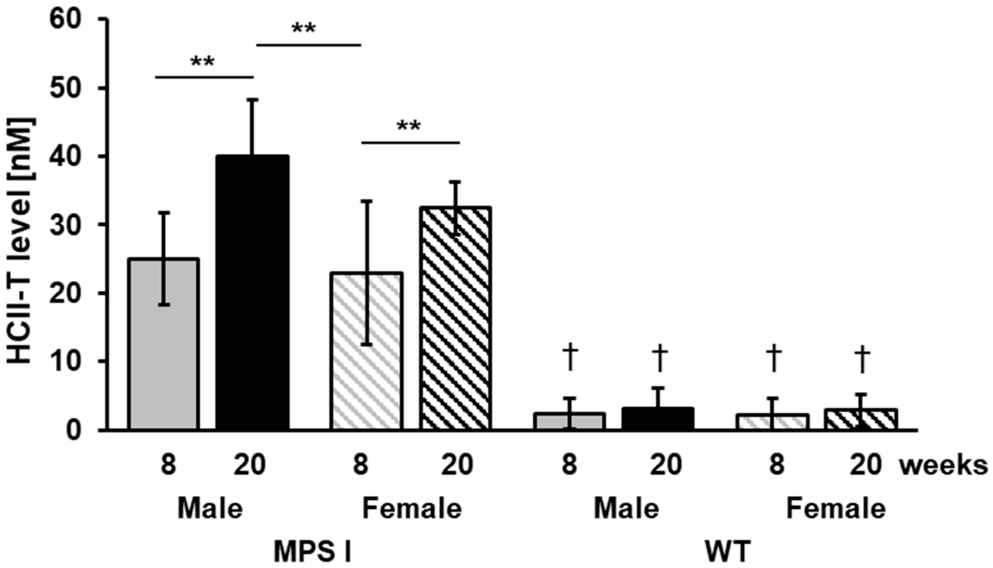

Figure S5. Comparison of serum levels of the heparin cofactor II-thrombin (HCII-T) complex in MPS I and wild-type (WT) animals. HCII-T complex levels were determined in the serum of untreated MPS I and WT mice of both sexes at 8 and 20 weeks of age. $N = 6$–18 animals per group. Significance: ** $p < 0.01$ between age groups and (Tukey's post-hoc after ANOVA) † $p < 0.05$ between age- and sex-matched MPS I and WT animals (Student's *t*-test).

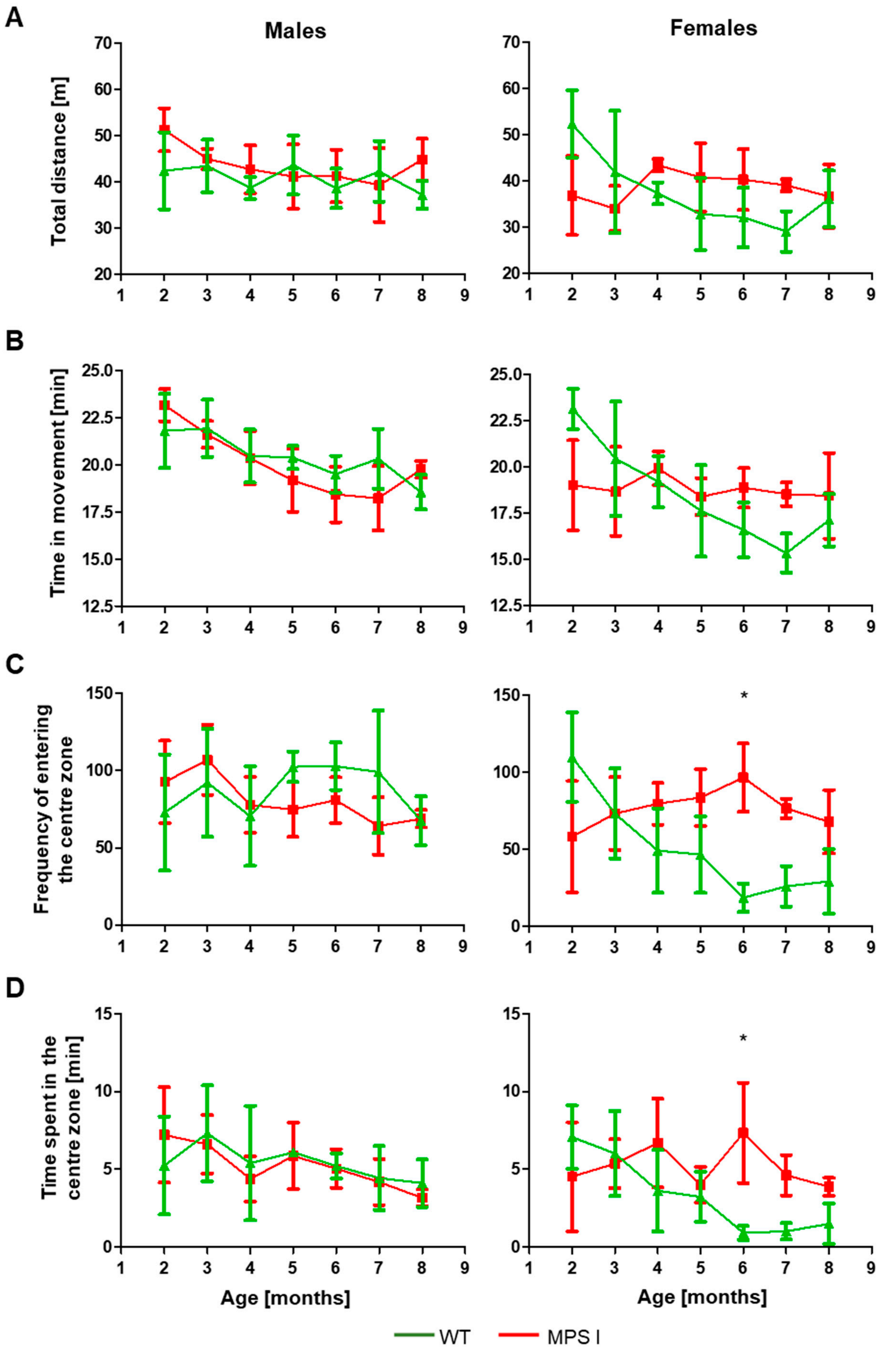

A
Males
Females
Total distance [m]
B
Time in movement [min]
C
Frequency of entering the centre zone
D
Time spent in the centre zone [min]
Age [months]
Age [months]
WT
MPS I

Figure S6. Preliminary behavioral analysis of MPS I and wild-type (WT) mice. Locomotor activity, exploration, and anxiety behavior of MPS I (red) and WT mice (green) of both sexes were assessed by the 30-minute open field test initially performed at two months of age, with subsequent testing every four weeks until they reached eight months of age. Locomotor activity and exploration were inferred based on the measurement of the total distance covered (**A**) and the total time an animal was in motion (**B**) during the test. Anxiety was inferred based on the frequency of animals entering the central zone of the arena (**C**) and the total time spent in the central zone (**D**) during the test. Results are presented as the mean ± standard deviation. $N = 3$ animals per group. Significance: * $p < 0.05$ (Student's *t*-test). The results indicated that younger female MPS I mice were less active and explored the environment less than their WT littermates did. This behavior remained unchanged regardless of the age of the animals. In contrast, younger female WT mice were more active and explored the environment more, but both behaviors decreased with age. The locomotor activity and anxiety behavior of male MPS I and WT mice were comparable, regardless of animal age.

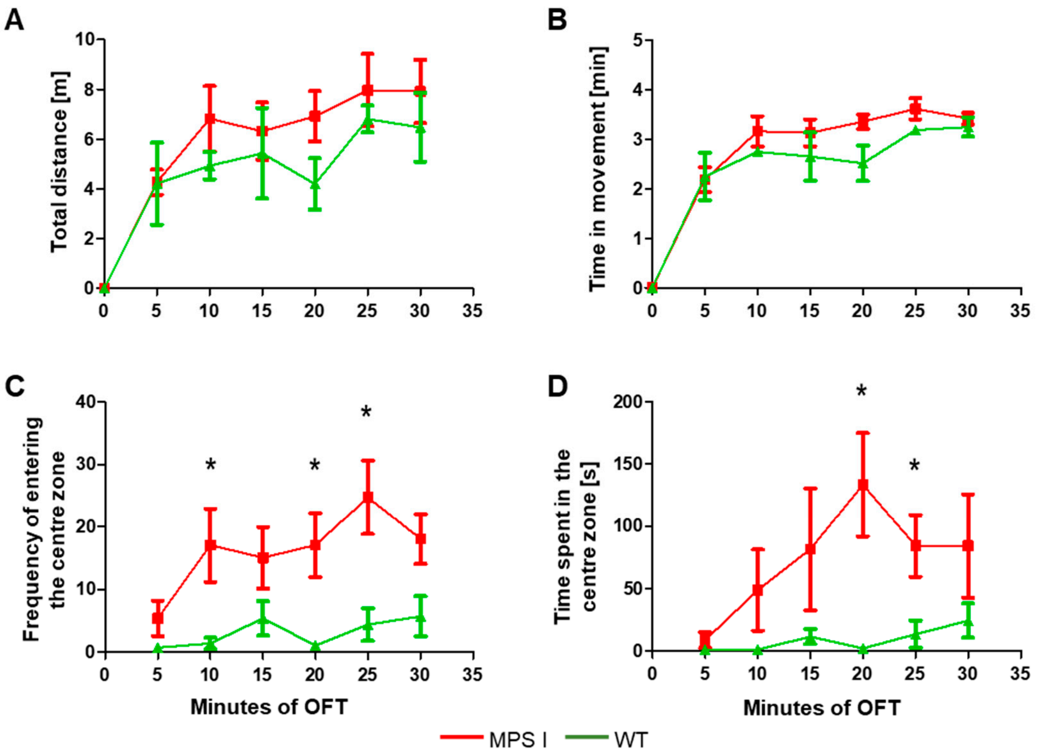

Figure S7. Behavior of six-month-old female MPS I and wild-type (WT) mice during a 30-minute open field test (OFT). The total distance covered (**A**), the total time an animal was in motion (**B**), the frequency of entering the central zone of the arena (**C**), and the total time spent in the central zone (**D**) were measured every 5 min during the test. MPS I mice are marked in red and WT mice in green. $N = 3$ animals per group. Results are presented as the mean ± standard deviation. Significance. * $p < 0.05$ between MPS I and WT animals (Student's *t*-test).

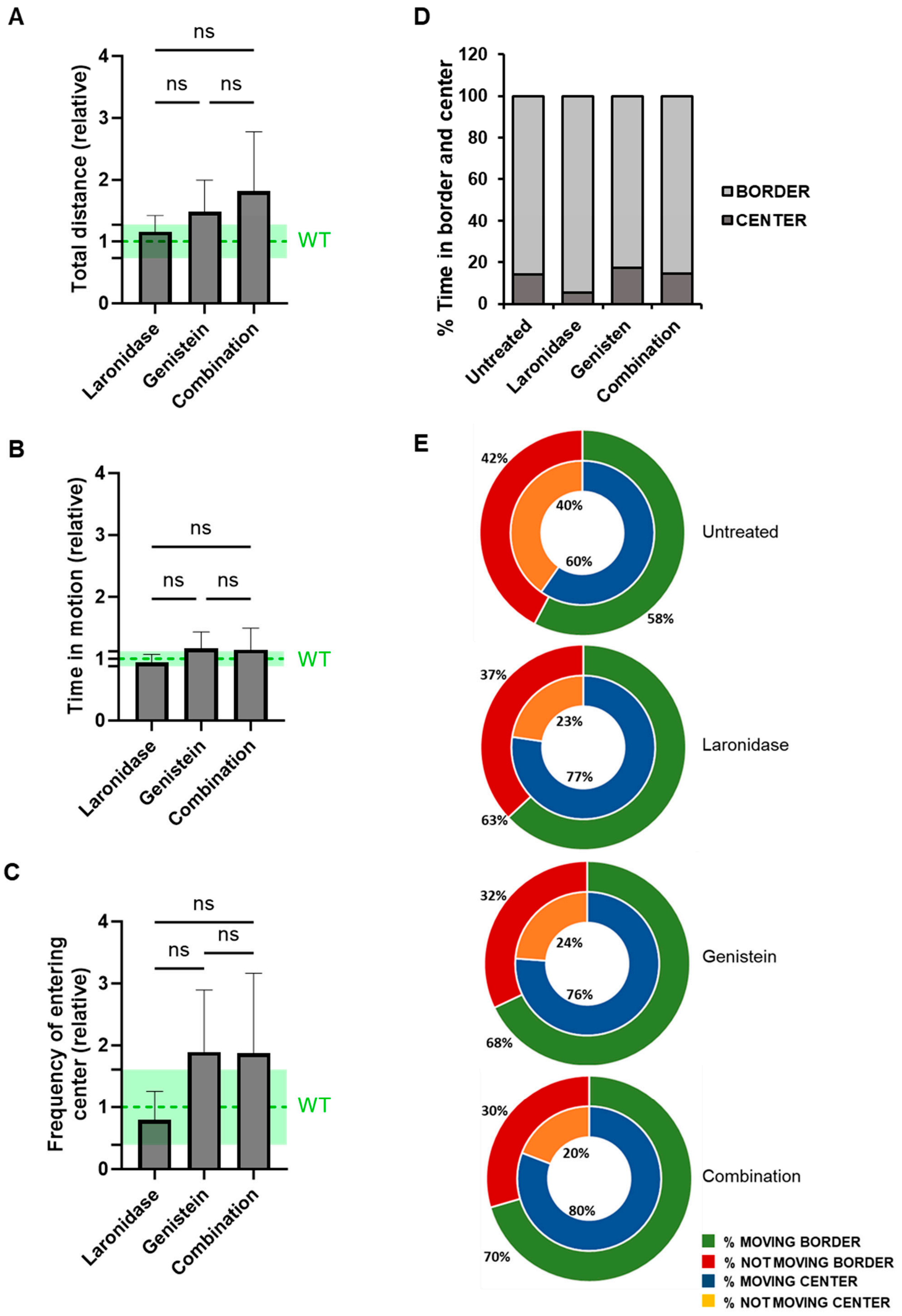
A
ns
ns
ns
Total distance (relative)
WT
Laronidase
Genistein
Combination
B
ns
ns
ns
Time in motion (relative)
WT
Laronidase
Genistein
Combination
C
ns
ns
ns
Frequency of entering center (relative)
WT
Laronidase
Genistein
Combination
D
% Time in border and center
BORDER
CENTER
Untreated
Laronidase
Genisten
Combination
E
42%
40%
60%
58%
Untreated
37%
23%
77%
63%
Laronidase
32%
24%
76%
68%
Genistein
30%
20%
80%
70%
Combination
% MOVING BORDER
% NOT MOVING BORDER
% MOVING CENTER
% NOT MOVING CENTER

Figure S8. Behavior of six-month-old female wild-type (WT) mice after treatment with laronidase, genistein, or combination therapy. Behavior was assessed during a 30-minute open field test for animals receiving 100 U/kg/week laronidase, 160 mg/kg/day genistein, or combination of both for 12 weeks. The results of (A) the total distance covered during the test, (B) total time that the animal remained in motion, and (C) frequency of entering the center zone of the arena were normalized to untreated WT animals (green dashed horizontal line with standard deviation shown as shaded field). Columns represent the mean ± standard deviation. N = 6 animals per group. No statistically significant differences were found between treated and untreated WT animals (Student's *t*-test), or between treatments (ANOVA); ns—not significant. (D) The percentage of time that mice spend in the center zone and the border of the arena and (E) the percentage of time that animals were moving (blue or green) or not moving (orange or red), in the center of the arena (inner ring) or on its periphery (outer ring), respectively. The results are presented as an average percentage of time for all animals in each group. The untreated control group consisted of age-matched female WT mice. N = 6 animals per group. No statistically significant differences were found between the treated and untreated WT animals (Student's *t*-test) or between the treatments (ANOVA).
